# A Study on the Level of Knowledge of Health Matters among Patients in a Rural Practice

**Published:** 1989-08

**Authors:** A. D. L. Austin

## Abstract

How much do patients know about everyday matters that can affect their health? A study of 200 patients attending a rural G.P. surgery was undertaken to find out.

It revealed some ignorance about the link between smoking and heart disease especially among female smokers (22% for this group). It showed a great deal of ignorance about alcohol in relation to health. Patients had a poor understanding of the relative alcohol content of different beverages, diseases related to excessive drinking and safe limits of alcohol consumption.


### A Study on the Level of Knowledge of Health Matters Among Patients in a Rural Practice

**A. D. L. Austin MB, ChB, DCH, DRCOG, GP Trainee**

### ABSTRACT
How much do patients know about everyday matters that can affect their health? A study of 200 patients attending a rural G.P. surgery was undertaken to find out.

It revealed some ignorance about the link between smoking and heart disease especially among female smokers (22% for this group). It showed a great deal of ignorance about alcohol in relation to health. Patients had a poor understanding of the relative alcohol content of different beverages, diseases related to excessive drinking and safe limits of alcohol consumption.

### INTRODUCTION
The word 'doctor' derives from the latin word 'docere' meaning 'to teach' and most doctors accept that they have an important role in the education of their patients. To do this it is useful to know in which areas patients are likely to lack knowledge. This study attempts to discover some of these areas.

### PATIENTS AND METHODS
Patients attending Langport Surgery in Somerset between August and November 1988 were invited to try a health questionnaire while they were waiting to see a doctor. The questionnaires were given out by receptionist staff and these consisted of 56 questions divided into 5 major categories. These were: diet and weight, exercise, alcohol, smoking and environment. The patients were asked to give their age, sex and state if they were a smoker or non-smoker. They were instructed to answer each question as true, false or don't know by ticking the appropriate column. A single mark was given for every correct answer.

Patients were asked to return their completed questionnaire to the receptionists, who would give them an answer sheet in exchange. Answers provided were phrased in a way which remind the reader of the original question.

The main advantages of the method used was the amount of time saved in the collection of data. If interviews were used to obtain information then 15 to 30 minutes might be needed per patient. Thus, to see 200 patients would take between 50 and 100 hours. Many patients might well be reluctant to give up 15 to 30 minutes of their time to be interviewed. There were plenty of patients willing to answer questions on a single sheet of paper, without help, while waiting to see a doctor. Handing out answer sheets was a useful way to educate patients that were interested.

There were some disadvantages of the method. For example, patients waiting to see a doctor are sometimes anxious or feeling quite unwell. As a result, some patients would not score as well as they might. In practice, however, such patients often decline to attempt the questionnaire at all. Secondly, without supervision, patients might not answer all the questions or provide all the information required of them. For example, in this study 14 patients failed to state their gender. Also patients are more likely to misinterpret questions if they cannot clarify points with anyone.

65 men and 121 women answered the questionnaire. The average age for men was 52 and for women 44. There were 15 men who smoked (23%) and 32 women (26%). 14 patients that did not state their gender, but answered all the questions, were included in the study. 18 patients that failed to complete the questionnaire were not included.

### RESULTS
The overall average score was 64%, males averaged 66% and females 63%. Age was not found to be a significant influence on scores obtained.

There were 20 questions on healthy eating and diseases caused by obesity. Average scores were high, 73% for females, 72% for males and 73% for all patients. With regard to dairy products, questions on cream were answered incorrectly by 8% of patients, questions on skimmed milk by 12% and questions on butter by 28.5%.

5 questions were on environment (air pollution and tetanus prophylaxis). Scores were high, 70% on average (74% for males and 69% for females).

There were 6 questions on exercise and scores were fairly high, 67% for females, 64% for males and 65% for all patients.

16 questions were on alcohol related diseases, the relative alcohol content of different drinks and safe levels of alcohol consumption. The average score was only 51% (56% for males and 49% for females). Both males and females scored very badly on questions comparing the alcohol content of different drinks. Both sexes gave fully correct answers in only 1.5% of replies. Questions about safe levels of drinking for men were answered correctly by 55% of males. Females correctly answered questions on safe levels of drinking for women in only 2% of replies. This may be explained by the fact that women were asked about wine drinking. Some questions showed that women tended to overestimate the alcohol content of wine and it was notable that women tended to underestimate safe levels of wine consumption. Another study, in London and Oxford, has shown the same thing (1).

There were 9 questions on smoking related diseases and trends in female smoking. Males averaged 63%, females 58% and all patients 59%. Scores were similar for smokers and non-smokers. The relationship between smoking and heart disease was not known by 15% of patients. It was only 4% of male non-smokers but reached 22% amongst female smokers.

### DISCUSSION

It was interesting that nearly 30% of patients classed butter as a healthy food. Perhaps this is because butter is often advertised using expressions such as "English and good for you" or "nothing added apart from a little salt". No mention is made of its high content of cholesterol and standard fats.

The most notable findings of the study concerned alcohol and smoking. A notable minority of patients did not know that smoking is a risk factor for coronary heart disease. The number was highest amongst female smokers, 22% for this group. Coronary heart disease is the greatest cause of premature death in Britain and smoking is an important preventable risk factor. Patient education in this matter is, therefore, of considerable importance and it is worth stressing the damage smoking can do to the heart in addition to lung disease. Last year cigarette consumption increased by 0.6%, probably related to a fall in the 'real price' of cigarettes (2). Women in particular have been smoking more in recent years. Educating women about the health risks of smoking, especially during pregnancy or when taking oral contraception, looks like being an increasingly important task for doctors.

The study showed that there was a great deal of ignorance about alcohol in relation to health. Patients knew little about diseases associated with excesive drinking. The scores for questions on the relative alcohol content of different drinks were extremely low. The questions on safe levels of drinking were better but still rather low. Alcohol related diseases affect large numbers of people in Britain today. Alcohol has become more accessible in the last 10 years. It is more available in supermarkets and shops and licencing hours have become longer. The consumption of wine and spirits has increased while the 'real price' has fallen (3). These factors mean that there is a considerable risk of alcohol related diseases increasing in the future. It is important that the general public should be aware of the facts about alcohol in relation to health. This study reveals that the scope for education in this area is considerable.

### ACKNOWLEDGEMENTS

Thanks to Drs M. Richards, D. Gibson, P. Nightingale, E. Nightingale and R. Obgers and their receptionist staff for their help with this study.

### REFERENCES

1. ANDERSON, P. and WALLACE, P. (1988). Safe limits of drinking: patients' views. *Br. Med. J.* **296**, 1707
2. TOWNSEND, J. (1989) Cigarette smoking on increase. *Br. Med. J.* **298** 1272.
3. SMITH, R. (1989). Call to rationalise alcohol taxation. *Br. Med. J.* **298**, 701.

Correspondence to: Dr. A. D. L. Austin, Pine Tree Cottage, Huish Episcopi, Langport, Somerset, TA10 9QT

